# Expression patterns of serum miR‐27a‐3p and activating transcription factor 3 in children with bronchial asthma and their correlations with airway inflammation

**DOI:** 10.1111/crj.13631

**Published:** 2023-06-29

**Authors:** Jingcai Wang, Lixin Yang, Peng Sun, Chunyan Guo, Yuzi Jin, Xiaoqing Jing

**Affiliations:** ^1^ Department of Pediatric The Affiliated Hospital of Chengde Medical University Chengde Hebei China

**Keywords:** activating transcription factor 3, airway inflammation, bronchial asthma, eosinophils, IL‐17, IL‐6, miR‐27a‐3p, TNF‐α

## Abstract

**Introduction:**

Bronchial asthma (BA) is a heterogeneous disease characterized by chronic airway inflammation. This study investigated the serum miR‐27a‐3p/activating transcription factor 3 (ATF3) expression in children with BA and their correlations with airway inflammation.

**Methods:**

Children with BA (*N* = 120) and healthy children (*N* = 108) were enrolled. Serum levels of interleukin (IL)‐17, IL‐6, tumor necrosis factor (TNF)‐α, immunoglobulin E (IgE), miR‐27a‐3p, ATF3, and the number of eosinophils (EOS) were measured using enzyme‐linked immunosorbent assay (ELISA), reverse transcription quantitative polymerase chain reaction (RT‐qPCR), and an automatic hematology analyzer. The correlations between miR‐27a‐3p and ATF3 and between miR‐27a‐3p/ATF3 and inflammation‐related factors were analyzed by the Pearson method. The diagnostic values of miR‐27a‐3p and ATF3 in BA were evaluated using receiver operating characteristic (ROC) curves. The influencing factors of BA were assessed using multivariate logistic regression. Finally, the targeting relation between miR‐27a‐3p and ATF3 was predicted and analyzed by TargetScan and Starbase databases, and dual‐luciferase assay.

**Results:**

There were significant differences in forced expiratory volume in 1 s (FEV1)% predicted and FEV1/forced vital capacity (FVC)%, serum levels of IgE, IL‐17, IL‐6, and TNF‐α, and EOS numbers between healthy children and BA children. Serum miR‐27a‐3p was negatively correlated with ATF3 and positively correlated with inflammation‐related factors in BA children. Serum ATF3 mRNA levels were negatively correlated with inflammatory factors in BA children. miR‐27a‐3p and ATF3 had good diagnostic values in BA children. FEV% predicted, IL‐6, TNF‐α, miR‐27a‐3p, and ATF3 were independent risk factors for BA. miR‐27a‐3p targeted ATF3.

**Conclusion:**

Serum miR‐27a‐3p was highly expressed, whereas ATF3 was poorly expressed in BA children, and they were significantly correlated with airway inflammation, had good diagnostic values in BA children, and were independent risk factors for asthma.

## Graphical abstract

Bronchial asthma (BA) is a heterogeneous disease characterized by chronic airway inflammation. This study investigated the serum miR‐27a‐3p/activating transcription factor 3 (ATF3) expression in children with BA and its correlation with airway inflammation.

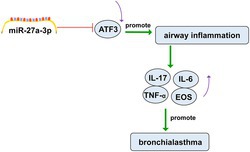

## 1 INTRODUCTION

Among chronic respiratory diseases, asthma is one of the most prevailing that affects over 300 million people across the globe.^1^ Bronchial asthma (BA) is a respiratory disease characterized by chronic airway inflammation, bronchial remodeling, and airway hyperresponsiveness.^2^ Its main clinical manifestations are wheezing, chest tightness, dyspnea, and recurrent cough, with complex etiology and pathogenesis.^3^ BA in children is a global public health issue that seriously affects their health, growth, and development, and morbidity and mortality are on the rise.^4^ At present, the predictive index of asthma is combined with domestic and foreign clinical experience to judge asthma with wheezing in children, but the diagnosis in children under 6 years of age is still challenging.^5^, ^6^ Underdiagnosis of asthma results in a delay in the optimal time for asthma treatment and may aggravate mild to severe refractory asthma.^7^ Therefore, there is a need to understand BA pathogenesis in children to improve the early diagnosis rate.

Asthmatic airway inflammation is mediated by many cell types, including T lymphocytes, macrophages, eosinophils (EOS), mast cells, and neutrophils.^8^, ^9^ A previous study has shown that the increased expression levels of pro‐inflammatory factors such as interleukin (IL)‐6, IL‐8, IL‐17, IL‐21, IL‐22, and tumor necrosis factor‐α (TNF‐α) in asthmatic patients are related to the development of BA.^10^, ^11^, ^12^ Besides, many inflammatory cells are infiltrated in the bronchial and lung tissues of asthmatic patients, and EOS with abnormally long lives are the main inflammatory components of allergic reactions.^13^ However, the specific mechanism behind these inflammatory cytokines triggering airway inflammation in BA has not yet been elucidated.

MicroRNAs (miRNAs) are small non‐coding RNAs in post‐transcriptional gene modulation, which are considered as major regulators of chronic lung diseases.^14^ Changes in miRNA abundance in inflammatory cells, lung tissue, and free blood circulation have been considered drivers and modifiers of diseases.^15^ Emerging research implies that serum miR‐27a is highly expressed in asthmatic patients and is involved in the occurrence of asthma by promoting airway smooth muscle cell proliferation and invasion by inhibiting mitogen‐activated protein kinase kinase 4 (MAP2K4).^16^ Moreover, tanshinone IIA ameliorates lipopolysaccharide (LPS)‐induced inflammation in the bronchial epithelial cell line BEAS‐2B by reducing miR‐27a.^17^ Besides, miR‐27a‐3p down‐regulation contributes to the occurrence of bronchiolitis obliterans, and it may be developed as a new therapeutic agent for bronchiolitis obliterans.^18^ However, serum miR‐27a‐3p expression in children with BA has rarely been reported.

Activating transcription factor 3 (ATF3) is an immune transcription factor regulated by the Toll‐like receptor (TLR) signaling, which in turn inhibits the transcription of genes encoding a variety of TLR‐driven proinflammatory mediators.^19^ Studies have reported that ATF3 has a significant correlation with asthma and can attenuate the inflammatory response in allergic airway disease.^20^, ^21^ What is more, ATF3 is a negative regulator of ovalbumin‐stimulated allergic inflammation in mice,^22^ and the lack of ATF3 induces airway hyperresponsiveness and increases pulmonary EOS in mice.^23^ In addition, miR‐27a‐3p targets ATF3 to diminish calcium deposition in vascular smooth muscle cells (VSMCs).^24^ However, serum miR‐27a‐3p/ATF3 expression patterns in children with BA and their association with airway inflammation have not yet been reported. This paper was to study the correlation between miR‐27a‐3p/ATF3 and airway inflammation in children with BA, hoping to provide theoretical references for new therapeutic targets for BA in children.

## 2 MATERIALS AND METHODS

### 2.1 Study subjects

BA children (*N* = 120) treated in The Affiliated Hospital of Chengde Medical University from January 2021 to December 2021 were enrolled, with 108 healthy children undergoing physical examination during the same period as the controls.

### 2.2 Inclusion criteria

(1) Diagnosed with BA based on the Global Initiative for Asthma guidelines (2016); (2) aged between 4 and 13 years; (3) no other infection except for the respiratory tract; (4) no malignant blood infection disease or tumor; (5) approved by the hospital ethics committee, children and their families voluntarily participated in the study and signed the consent form; (6) control children had no family or personal history of allergies or asthma.

### 2.3 Exclusion criteria

(1) Children with cardiogenic asthma, bronchogenic lung cancer, tracheal intimal lesions, and allergic pulmonary infiltration; (2) taking glucocorticoids within 1 month before the study; (3) taking anti‐asthma medications such as bronchodilators within 1 week; (4) with other systemic diseases throughout the body; (5) with neoplasms, hematological diseases, lower respiratory tract, and systemic infectious diseases.

### 2.4 Data and sample collection

Baseline data, such as age, gender, family history of asthma, and body mass index (BMI), were recorded. The percent predicted forced expiratory volume in 1 s (FEV1% predicted) and the percentage of FEV in 1 s to forced vital capacity (FEV1/FVC%) were measured by a pulmonary function detector (M&B Electronic Instruments, Beijing, China). Fasting elbow venous blood (3 mL) was collected from two groups of children in the morning. The serum was separated by centrifugation and stored at −70°C.

### 2.5 Enzyme‐linked immunosorbent assay (ELISA)

Serum levels of IL‐17 (RAB0262), IL‐6 (RAB0306), and TNF‐α (RAB0489) were measured according to the ELISA kit (Sigma‐Aldrich, MO, USA) instructions. The serum level of IgE was measured according to the product instructions of the Human IgE ELISA Kit (PI479) (Beyotime, Shanghai, China).

### 2.6 EOS count

Blood cell counts were determined using an automated hematology analyzer (Beckman Coulter, CA, USA), and EOS counts in blood cells were calculated. The process was repeated thrice to get the mean value.

### 2.7 Reverse transcription quantitative polymerase chain reaction (RT‐qPCR)

Total RNA was extracted from serum using TRIzol (Thermo Fisher, MA, USA) and reverse transcribed into cDNA according to the instructions of the reverse transcriptase kits (Takara, Dalian, China). RT‐qPCR was conducted on an ABI 7500 rapid real‐time PCR system (ABI, CA, USA) using SYBR Green Real‐Time PCR Master Mix (Takara) and miScript SYBR Green PCR kits (Qiagen, Germany). The PCR conditions were as follows: 40 cycles of 94°C for 30 s, 60°C for 30 s, and 72°C for 30 s. With U6 as the internal reference gene, miR‐27a‐3p expression was assessed, and with glyceraldehyde 3‐phosphate dehydrogenase (GAPDH) as the internal reference gene, ATF3 expression was assessed. The primer sequences are shown in Table 1, and three replicates were created per sample. The relative levels of the targeted mRNAs were calculated with the 2^−ΔΔCt^ method.

**TABLE 1:** Primer sequences.

Gene	Forward 5′‐3′	Reverse 5′‐3′
miR‐27a‐3p	ATGGTTCGTGGGTTCACA	GTGGCTAAGTTCCGACG
U6	ATACAGAGAAGATTAGCATGGCCCCTG	ACACGCAAATTCGTGAAGCGTTCCATATTT
ATF3	CCTCTGCGCTGGAATCAGTC	TTCTTTCTCGTCGCCTCTTTTT
GAPDH	ATCATCCCTGCATCCACT	ATCCACGACGGACACATT

Abbreviations: ATF3, activating transcription factor 3; GAPDH, glyceraldehyde 3‐phosphate dehydrogenase.

### 2.8 Dual‐luciferase reporter assay

TargetScan database (https://www.targetscan.org/vert_71/) and Starbase database (http://starbase.sysu.edu.cn/index.php) prediction analysis revealed the presence of binding sites between miR‐27a‐3p and ATF3. The complementary and mutant sequences of miR‐27a‐3p and ATF3 were cloned onto the pmir‐GLO luciferase reporter vector plasmid (Promega, WI, USA) to construct ATF3‐WT and the corresponding ATF3‐MUT. ATF3‐WT and ATF3‐MUT were co‐transfected with mimics NC or miR‐27a‐3p mimics into HEK293T cells using Lipofectamine™ 2000 (Invitrogen, CA, USA). After 48 h, luciferase activity was measured using a dual‐luciferase system (Promega).

### 2.9 Statistical analysis

SPSS 21.0 (IBM Corp., Armonk, NY, USA) and GraphPad Prism 8.01 software (GraphPad Software, San Diego, CA, USA) were adopted for data analysis and plotting. Count data were shown as the number of cases and percentage and compared using a chi‐squared test. Measurement data were depicted as mean ± standard deviation (SD) and analyzed by the *t*‐test for pairwise comparisons and one‐way analysis of variance (ANOVA) for multi‐group comparisons. The Pearson method was utilized to analyze the correlation between the relative levels of miR‐27a‐3p and ATF3 mRNA and the correlations between miR‐27a‐3p/ATF3 and IL‐17, IL‐6, TNF‐α, and EOS. The receiver operating characteristic (ROC) curve was plotted to analyze the diagnostic value of miR‐27a‐3p/ATF3. Multivariate logistic regression analysis was utilized to analyze the influencing factors of BA.

## 3 RESULTS

### 3.1 Clinical baseline characteristics of the enrolled population

A total of 120 children with BA were included, with 108 healthy children as controls. The baseline clinical data of all participants were statistically analyzed. Table 2 showed no significant differences in gender, age, family history of asthma, and BMI between healthy people and BA children (*p* > 0.05); however, there were significant differences in the pulmonary function indexes FEV1% predicted and FEV1/FVC%, serum levels of IgE, IL‐17, IL‐6, and TNF‐α, and the number of EOS in blood cells (all *p* < 0.05).

**TABLE 2:** Clinical baseline characteristics of the enrolled population.

Parameters	Normal (*N* = 108)	BA (*N* = 120)	*p* value
Gender (male/female)	65/43	70/50	0.7763
Age (years)	9.39 ± 2.75	9.18 ± 2.25	0.5270
Family history of asthma (%)	16 (14.81%)	23 (19.17%)	0.3836
BMI	16.21 ± 1.52	16.71 ± 1.69	0.0626
FEV1% predicted	94.37 ± 5.53	87.35 ± 4.71	<0.0001
FEV1/FVC%	90.08 ± 4.11	84.27 ± 4.19	<0.0001
IgE (IU/mL)	98.65 ± 34.18 (32.00–233.61)	172.25 ± 40.59 (51.63–266.01)	<0.0001
IL‐17 (pg/mL)	67.97 ± 12.38 (35.99–111.00)	90.39 ± 10.78 (69.44–140.00)	<0.0001
IL‐6 (pg/mL)	103.32 ± 19.72 (35.00–189.55)	165.50 ± 14.53 (127.62–193.66)	<0.0001
TNF‐α (pg/mL)	91.23 ± 11.89 (69.18–125.10)	142.72 ± 16.13 (97.26–176.93)	<0.0001
EOS (× 10^9^/L)	0.63 ± 0.41 (0.29–2.69)	1.39 ± 0.38 (0.36–2.62)	<0.0001

*Note*: Gender and family history of asthma were analyzed by chi‐square method, and the rest were analyzed by *t*‐test.

Abbreviations: EOS, eosinophils; FEV1% predicted, percent predicted forced expiratory volume in 1 s; FEV1/FVC%, percentage of forced expiratory volume in 1 s to forced vital capacity; IgE, immunoglobulin E; IL‐17, interleukin‐17; IL‐6, interleukin‐6; TNF‐α, tumor necrosis factor‐α.

### 3.2 Correlation between serum miR‐27a‐3p and ATF3 mRNA levels in BA children

**FIGURE 1:**
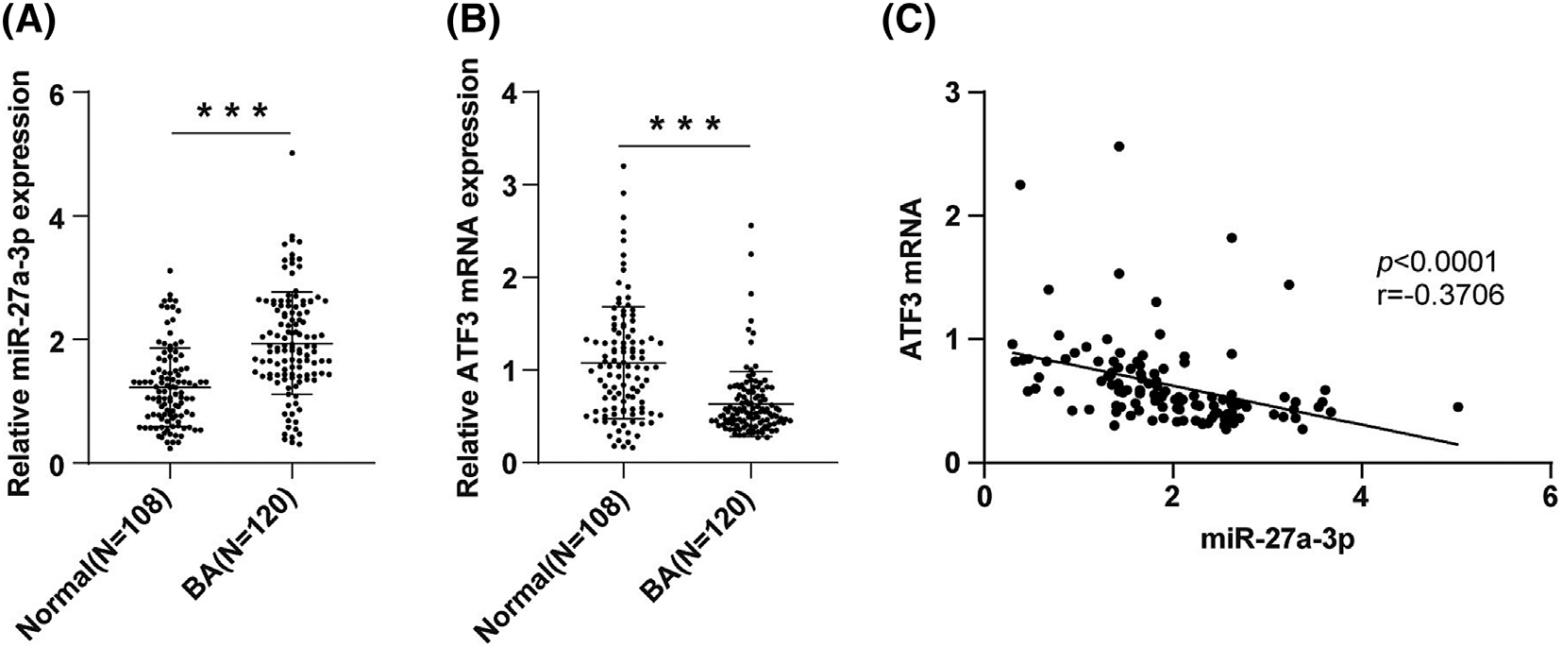
miR‐27a‐3p was negatively correlated with activating transcription factor 3 (ATF3) mRNA levels in bronchial asthma (BA) children. (A) and (B) The serum levels of miR‐27a‐3p and ATF3 mRNA were determined by reverse transcription quantitative polymerase chain reaction (RT‐qPCR). (C) Pearson correlation between serum miR‐27a‐3p and ATF3 mRNA levels in children with BA.

miR‐27a is associated with the pathogenesis of asthma,^16^ and ATF3 can attenuate the inflammatory response associated with allergic airway disease.^21^ Therefore, we detected the serum levels of miR‐27a‐3p and ATF3 mRNA by RT‐qPCR, which exhibited that the serum levels of miR‐27a‐3p in BA children were visibly higher (*p* < 0.001) (Figure 1A), whereas the serum ATF3 mRNA levels were notably lower in BA children than healthy children (*p* < 0.001) (Figure 1B). Additionally, we analyzed the correlation of serum miR‐27a‐3p with ATF3 mRNA levels by Pearson analysis, which manifested that miR‐27a‐3p and ATF3 mRNA levels were negatively correlated (*p* < 0.0001, *r* = −0.3706) (Figure 1C).

### 3.3 Correlation of serum miR‐27a‐3p/ATF3 with airway inflammation in BA children

**FIGURE 2:**
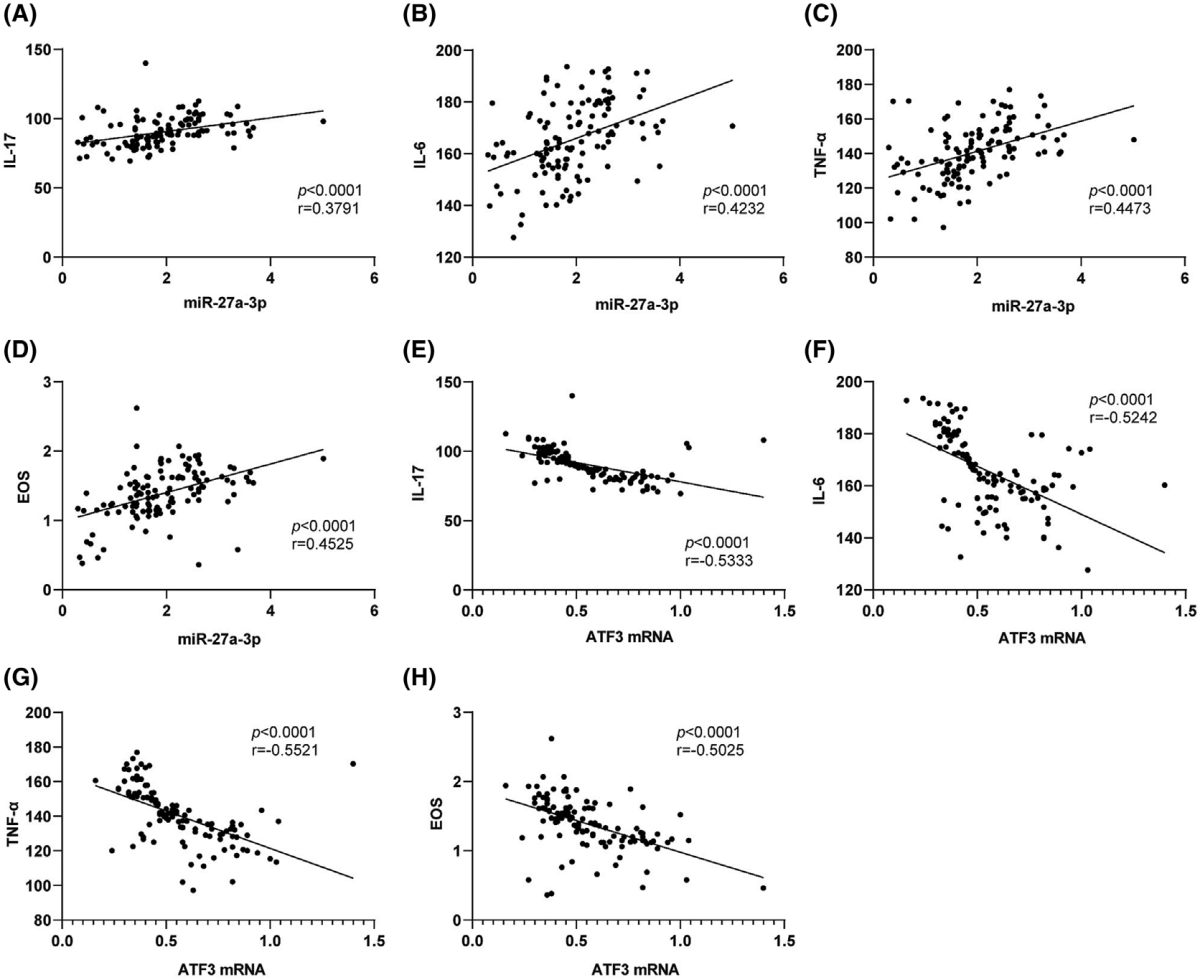
Correlation between miR‐27a‐3p/activating transcription factor 3 (ATF3) and interleukin (IL)‐17, IL‐6, tumor necrosis factor‐α (TNF‐α), and eosinophils (EOS). (A–D) Pearson correlation between serum miR‐27a‐3p and IL‐17, IL‐6, TNF‐α, and EOS in children with bronchial asthma (BA); (E–H) Pearson correlation between serum ATF3 mRNA levels and IL‐17, IL‐6, TNF‐α, and EOS.

To further evaluate the correlation of serum miR‐27a‐3p/ATF3 with inflammation in BA children, Pearson analysis was adopted. The results revealed that serum miR‐27a‐3p in children with BA was positively correlated with IL‐17, IL‐6, TNF‐α, and EOS (*p* < 0.0001, *r* = 0.3791; *p* < 0.0001, *r* = 0.4232; *p* < 0.0001, *r* = 0.4473; *p* < 0.0001, *r* = 0.4525) (Figure 2A–D); serum ATF3 mRNA levels in BA children were negatively correlated with these factors (*p* < 0.0001, *r* = −0.5333; *p* < 0.0001, *r* = −0.5242; *p* < 0.0001, *r* = −0.5521; *p* < 0.0001, *r* = −0.5025) (Figure 2E–H).

### 3.4 Diagnostic values of miR‐27a‐3p/ATF3 in children with BA

**FIGURE 3:**
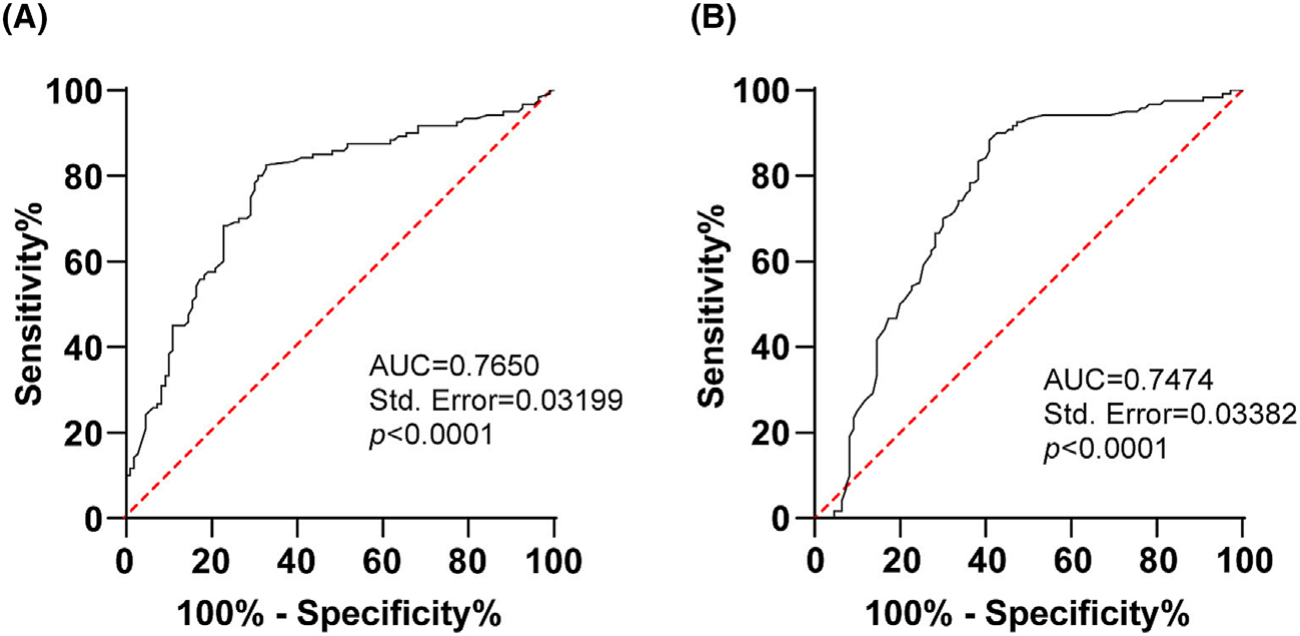
Receiver operating characteristic (ROC) curve analysis of miR‐27a‐3p/activating transcription factor 3 (ATF3) in the diagnosis of bronchial asthma (BA) children. (A) ROC curve analysis of miR‐27a‐3p in the diagnosis of BA children; (B) ROC curve analysis of ATF3 in the diagnosis of BA children.

ROC curve analysis was performed between healthy children and BA children to further assess the diagnostic value of miR‐27a‐3p/ATF3 in BA. As a result, the area under the ROC curve (AUC) of miR‐27a‐3p in diagnosing BA in children was 0.7650 and the cut‐off value was 1.325, with 82.5% sensitivity and 67.27% specificity (Figure 3A). The AUC of ATF3 in the diagnosis of BA in children was 0.7474 and the cut‐off value was 0.8850, with 88.33% sensitivity and 59.09% specificity (Figure 3B). Briefly, both miR‐27a‐3p and ATF3 had good diagnostic values for BA children.

### 3.5 Logistic regression analysis of factors affecting the occurrence of BA

Next, we assessed the factors that influence the occurrence of BA. Taking the occurrence of BA as the dependent variable, we included FEV1% predicted, FEV1/FVC %, IgE, IL‐17, IL‐6, TNF‐α, EOS, miR‐27a‐3p, and ATF3 with *p* < 0.0001 in Table 3 as the independent variables for multivariate logistic regression analysis. The results demonstrated that FEV% predicted, IL‐6, TNF‐α, miR‐27a‐3p, and ATF3 were independent risk factors for BA (all *p* < 0.05).

**TABLE 3:** Logistic regression analysis of factors affecting the occurrence of bronchial asthma (BA).

Factor	*p* value	OR	95% CI
FEV1% predicted	0.041	0.635	0.398–1.013
FEV1/FVC%	0.056	0.461	0.230–0.923
IgE	0.535	1.012	0.974–1.051
IL‐17	0.119	0.895	0.778–1.029
IL‐6	0.030	1.095	1.009–1.190
TNF‐α	0.015	1.485	1.081–2.040
EOS	0.274	5.463	0.261–114.245
miR‐27a‐3p	0.031	0.024	0.001–0.709
ATF3	0.033	462.214	1.619–131996.139

Abbreviations: ATF3, activating transcription factor 3; EOS, eosinophils; FEV1% predicted, percent predicted forced expiratory volume in 1 s; FEV1/FVC%, percentage of forced expiratory volume in 1 s to forced vital capacity; IL‐17, interleukin‐17; IL‐6, interleukin‐6; TNF‐α, tumor necrosis factor‐α.

### 3.6 miR‐27a‐3p targeted ATF3

**FIGURE 4:**
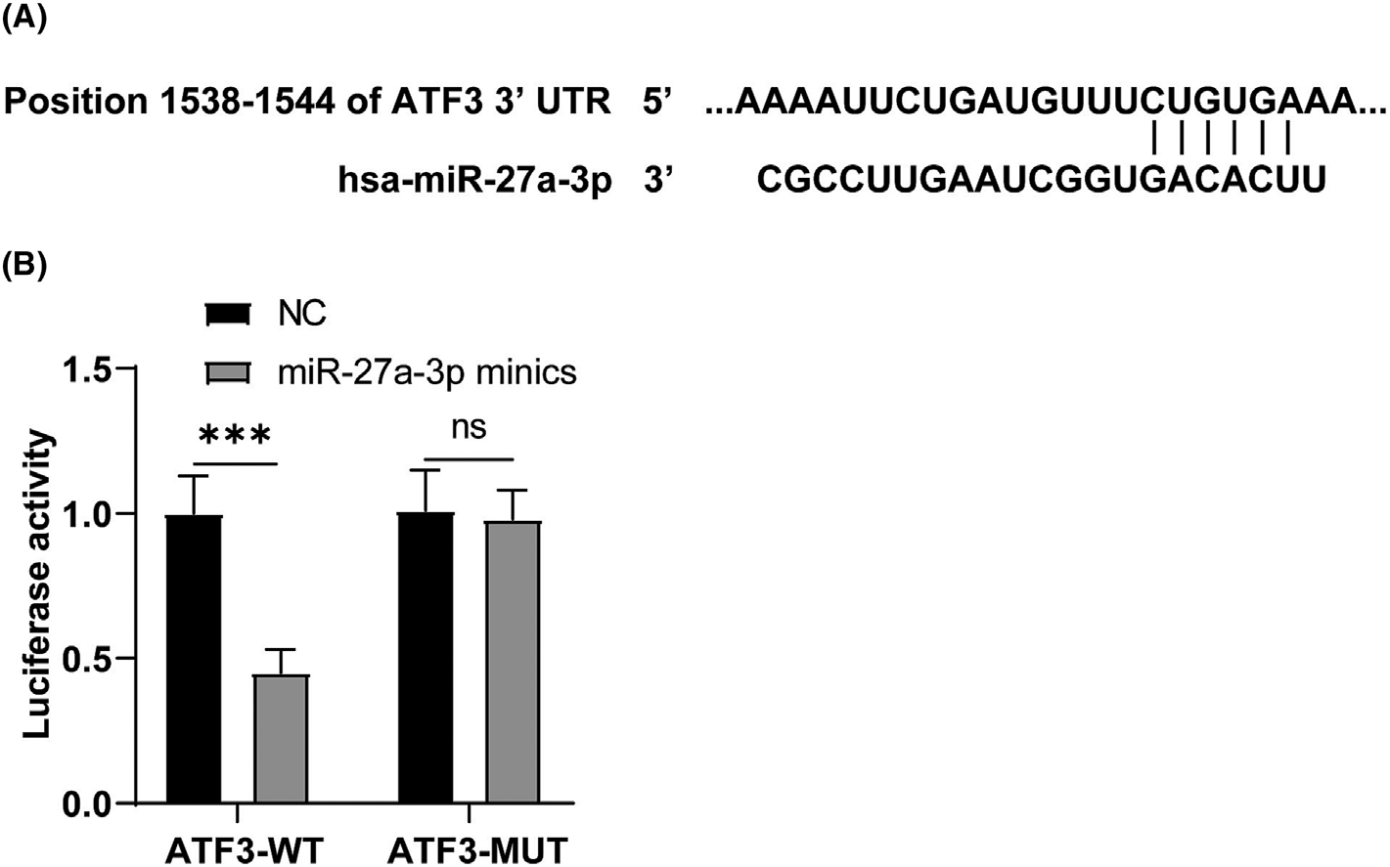
miR‐27a‐3p targeted activating transcription factor 3 (ATF3). (A) The target binding sites between miR‐27a‐3p and ATF3 were predicted by the TargetScan database and the Starbase database; (B) Dual‐luciferase reporter assay. Measurement data were expressed as mean ± SD. One‐way analysis of variance (ANOVA) was used for data comparison among multiple groups, followed by the Tukey's test. *** *p* < 0.001.

According to the predicted analysis of TargetScan and Starbase databases, there were targeted binding sites between miR‐27a‐3p and ATF3, and the target binding site sequences were consistent (Figure 4A). Dual‐luciferase assay displayed that luciferase activity was visibly reduced in human bronchial epithelial cells (HBECs) with miR‐27a‐3p mimics and ATF3‐WT (*p* < 0.001). However, there was no notable change in luciferase activity in HBECs with miR‐27a‐3p mimics and ATF3‐MUT (*p* = 0.8818) (Figure 4B). In short, ATF3 is the target gene of miR‐27a‐3p.

## 4 DISCUSSION

BA is a chronic inflammatory disease with a complex course.^25^ To date, efforts to identify biomarkers to predict asthma severity, course, and response to treatment have not been very successful.^26^ miRNAs have been found to have potential as biomarkers in patients with asthma symptoms in childhood, which may be helpful treatment targets for childhood asthma.^27^ Moreover, ATF3 has been discovered to be associated with the risk of asthma.^23^ Our findings illustrated that in the serum of children with BA, miR‐27a‐3p targets ATF3 and inhibits ATF3 levels, promotes airway inflammation, and upregulates the levels of IL‐17, IL‐6, TNF‐a, and EOS, thereby promoting the occurrence of asthma.

The pulmonary function indexes FEV1% predicted and FEV1/FVC%, as vital regulatory indexes for the assessment, treatment, and severity monitoring of BA, have always been the focus of respiratory physicians.^28^ Serum inflammatory factors may reflect the severity of BA.^29^ Overwhelming evidence indicates that the lung function of BA patients decreased gradually, whereas serum IL‐6, IL‐17, IgE, and TNF‐α were enhanced.^29^, ^30^, ^31^, ^32^ Meanwhile, as important cells involved in the pathogenesis of chronic airway inflammation in asthma, the increase of EOS is correlated with the exacerbation of BA.^33^, ^34^ Consistent with previous studies, the present study uncovered that in comparison with healthy children, FEV1% predicted and FEV1/FVC% were reduced in BA children, whereas inflammatory factors (IL‐6, IL‐17, IgE, and TNF‐α) and EOS were up‐regulated, which may be related to lung dysfunction and lung inflammation in BA patients.

There are numerous studies on the relationship between miRNAs and BA in children at home and abroad.^35^ Huo et al. reported that miR‐181b‐5p in epithelial cells and plasma is a potential biomarker of airway eosinophilia, which participates in eosinophil airway inflammation by regulating the expression of pro‐inflammatory cytokines by targeting SPP1.^36^ Additionally, miR‐27a has been revealed to be highly expressed in asthmatic patients,^16^ whereas whether miR‐27a‐3p affects airway inflammation in children with BA remains unclear. ATF3, a negative regulator of inflammation, can inhibit the malignant transformation of bronchial epithelial cells by alleviating inflammation^37^ and is reduced in bronchoalveolar lavage fluid from patients infected with *Mycobacterium tuberculosis*.^38^ As expected, our study revealed a high expression of serum miR‐27a‐3p and low levels of ATF3 mRNA in children with BA, suggesting that they may be used as objective indicators to distinguish BA children from healthy children. According to ROC curve analysis, our subsequent experiment identified that miR‐27a‐3p (AUC = 0.7650, cut‐off value = 1.325) and ATF3 (AUC = 0.7474, cut‐off value = 0.8850) could distinguish BA children from healthy children in our study population. Our study illustrated for the first time that miR‐27a‐3p and ATF3 have good diagnostic values in children with BA and may become new diagnostic markers for BA. It has been reflected that the reduction of miR‐27a‐3p increased ATF3 levels in VSMCs.^24^ Similarly, we discovered a negative correlation between miR‐27a‐3p and ATF3 mRNA levels. Moreover, a dual‐luciferase assay revealed that ATF3 is the target of miR‐27a‐3p. Altogether, we identified that miR‐27a‐3p targeted ATF3, which enriches the theoretical knowledge of the regulatory mechanisms of BA.

Pro‐inflammatory cytokines are the main factors that cause inflammation, and their activation is the key to accelerating disease progression.^39^ Accordingly, Pearson analysis unveiled that serum miR‐27a‐3p was positively correlated with inflammatory factors and EOS levels in BA children, and serum ATF3 mRNA levels were negatively correlated with their levels. Consistently, miR‐27a‐3p was positively correlated with renal inflammatory dysfunction, cell proliferation, and apoptosis.^40^ Moreover, ATF3 can inhibit the release of inflammatory factors TNF‐α, IL‐1β, IL‐6, and IL‐18 induced by *Mycoplasma pneumonia*.^41^ Therefore, we can conclude that both high serum miR‐27a‐3p levels and low ATF3 mRNA levels may promote the production of inflammation in BA children. A previous study reported that IL‐4, FEV1/FVC, and peak expiratory flow levels were independent risk factors for the prognosis of children with moderate to severe asthma.^42^ Our experimental data manifested that FEV% predicted, IL‐6, TNF‐α, miR‐27a‐3p, and ATF3 were independent risk factors for BA.

In conclusion, serum miR‐27a‐3p and ATF3 in children with BA are closely related to airway inflammation, and it also has a certain clinical value in the early diagnosis of BA. Nevertheless, the present study possesses the following limitations. Firstly, the number of cases collected is limited. The regulatory mechanisms of miR‐27a‐3p/ATF3 and inflammatory factors in BA children still need to be studied. Furthermore, multiple genes, such as ATF3, activation‐induced cytidine deaminase (AICDA), thymic stromal lymphopoietin (TSLP), and endothelin receptor type A (EDNRA), are associated with asthma.^43^, ^44^, ^45^ Through predictive analysis of the TargetScan database, we found that miR‐27a‐3p might have a targeted binding relationship with ATF3, AICDA, TSLP, and EDNRA. In addition, according to a previous study, ATF3 has a significant correlation with asthma and is associated with the risk of asthma.^23^, ^46^ However, because of limited time and funding, in this study, we focused on the expression levels of ATF3 and miR‐27a‐3p in the serum of children with BA and their correlation with airway inflammation. We also verified the existence of a targeted binding relationship between miR‐27a‐3p and ATF3 by dual‐luciferase reporting assay. In future research, we will further explore the relationship between miR‐27a‐3p and AICDA, TSLP, EDNRA, and other asthma‐related genes and their expression levels and clinical roles in the serum of children with BA. Therefore, future studies would also need to take into account the limitations of the current project.

## AUTHOR CONTRIBUTIONS

Jingcai Wang and Xiaoqing Jing are the guarantors of the integrity of the entire study. Jingcai Wang and Peng Sun contributed to the study concepts and clinical studies. Jingcai Wang contributed to the study design, experimental studies, data analysis, manuscript preparation, and manuscript editing. Chunyan Guo contributed to the definition of intellectual content. Jingcai Wang, Lixin Yang, and Peng Sun contributed to the literature research and data acquisition. Jingcai Wang and Yuzi Jin contributed to the statistical analysis. Xiaoqing Jing contributed to the manuscript review. All authors read and approve the final manuscript.

## CONFLICT OF INTEREST STATEMENT

All authors declare that there is no conflict of interest in this study.

## ETHICS APPROVAL

This study was ratified by the Academic Ethics Committee of The Affiliated Hospital of Chengde Medical University. All participants were informed of the study purpose and signed informed consent before sample collection. All procedures were strictly implemented according to the *Declaration of Helsinki*.

## Data Availability

The data that support the findings of this study are available from the corresponding author upon reasonable request.
